# Imprinted Polymers as Synthetic Receptors in Sensors for Food Safety

**DOI:** 10.3390/bios11020046

**Published:** 2021-02-11

**Authors:** Rocio Arreguin-Campos, Kathia L. Jiménez-Monroy, Hanne Diliën, Thomas J. Cleij, Bart van Grinsven, Kasper Eersels

**Affiliations:** Sensor Engineering Department, Faculty of Science and Engineering, Maastricht University, P.O. Box 616,6200 MD Maastricht, The Netherlands; r.arreguincampos@maastrichtuniversity.nl (R.A.-C.); k.jimenezmonroy@maastrichtuniversity.nl (K.L.J.-M.); hanne.dilien@maastrichtuniversity.nl (H.D.); thomas.cleij@maastrichtuniversity.nl (T.J.C.); bart.vangrinsven@maastrichtuniversity.nl (B.v.G.)

**Keywords:** imprinted polymers, food safety, biomimetic sensor

## Abstract

Foodborne illnesses represent high costs worldwide in terms of medical care and productivity. To ensure safety along the food chain, technologies that help to monitor and improve food preservation have emerged in a multidisciplinary context. These technologies focus on the detection and/or removal of either biological (e.g., bacteria, virus, etc.) or chemical (e.g., drugs and pesticides) safety hazards. Imprinted polymers are synthetic receptors able of recognizing both chemical and biological contaminants. While numerous reviews have focused on the use of these robust materials in extraction and separation applications, little bibliography summarizes the research that has been performed on their coupling to sensing platforms for food safety. The aim of this work is therefore to fill this gap and highlight the multidisciplinary aspects involved in the application of imprinting technology in the whole value chain ranging from IP preparation to integrated sensor systems for the specific recognition and quantification of chemical and microbiological contaminants in food samples.

## 1. Introduction

With the increasing globalization of food trade and supply, food safety is an issue able to trigger health emergencies with international impact, for example the Listeriosis outbreak in South Africa (2017–2018), Europe (2018) and Spain (2019) [1], which required a multinational collaboration. The World Health Organization estimates about 600 million cases of foodborne diseases (FBD) every year [2]. FBD can range from diarrhea to cancers and affects nearly 1 out of 10 people globally [3]. Apart from the impact on human well-being, economic costs related to FBD include productivity losses, treatment of the diseases, domestic market disruption and costumer product avoidance [4]. Therefore, strict controls and tools able to assess that all foods remain safe, wholesome and fit for human consumption are essential. 

A hazard, in terms of food safety, is any agent that can cause an adverse health effect, and can be classified by its physical, chemical or biological nature [5]. The prevention and accurate identification of these agents in the food supply chain is fundamental for avoiding FBD. Food needs to go through a multistep process with a variable number of actors from production to consumption [6]. Such a dynamic system requires adequate technologies for the detection of chemical (e.g., pesticides or drug resides) and biological contaminants (bacteria, viruses and parasites). Analytical methods used for detecting chemical agents in food are gas and liquid chromatography, often in combination with mass spectrometry [7]. Biological detection, on the other hand, can be performed using traditional microbiological culturing, electrophoresis and polymerase chain reaction-based methods. These techniques can be costly and/or time-consuming, emphasizing the need for reliable, cost-effective and fast detection technologies [8]. Sensors, which possess these characteristics, are also suitable for detecting both chemical and biological agents and could be adapted to the requirements of each step of the food supply chain [9].

Sensing platforms can be described as two-component systems consisting of: (*i*) a recognition element, able to bind and provide a response when the targeted analyte is present; and (*ii*) a transducer, which converts the interactions derived from the binding of the recognition element with the target into analytical signals [10,11]. Sensors based on biological recognition elements (e.g., enzymes, nucleic acids, antibodies, cells, etc.) are the most researched not only in terms of food safety, but also for medical diagnostics and environmental monitoring [12]. Biological receptors, however, are fragile; require carefully regulated operational conditions such as pH values, ionic strength or temperature; and have a limited shelf life [13]. Biomimetic or synthetic receptors, as alternatives, are able to attain the affinity of biological receptors and overcome stability and durability issues [14,15,16].

Imprinted polymers (Ips) have attracted attention due to their simplicity in preparation and affinity attained as recognition element. The versatility of Ips enables detection of small molecules (Molecularly Imprinted Polymers, MIPs) [17,18,19] and whole cells (Surface Imprinted Polymers, SIPs) [20,21,22]. Furthermore, they can be coupled to different transducing elements such as microgravimetric [23,24,25], optical [26,27], thermal [28,29,30] and electrochemical [31,32,33,34] read-out platforms, among others.

FBD are certainly foreseen to rise in the upcoming years due to the increased complexity of food supply chains. Factors such as the globalization in food trade, changes of patterns in human consumption and climate change are only some of the underlying reasons more diseases related to harmful organisms and chemicals are expected [27,28]. Even though biomimetic sensors are suitable to detect these agents and have proven to possess several advantages over biosensors, their specific application in food safety has barely been reviewed [35,36].

This work covers the types of imprinted polymers that have been developed for the detection of chemical and biological hazards in food, their preparation strategies, coupling to different readout platforms and application in real food samples.

## 2. Imprinted Polymers

As mentioned above, biosensors are receptor–transducer devices defined as “chemical sensors in which the recognition system utilizes a biological element” [37]. This system involves specific receptor–ligand interactions, where complementarity of geometry and chemical functionality (e.g., covalent interactions, hydrogen bonds, van der Waals forces, etc.) define selectivity [38]. Decades of efforts using natural recognition elements as inspiration have resulted in diverse strategies for preparing synthetic receptors that mimic the function of biological receptors [39,40,41]. Furthermore, with the first report of artificial antibodies [42], the interest in molecularly imprinted polymers (MIPs) increased [15,43,44]. Imprinted polymers are excellent candidates for bio(mimetic) sensing platforms due to their simplicity in preparation, attained affinity and stability compared to their natural counterparts [15,17,38,44,45,46,47,48]. Moreover, imprinted polymers are robust materials that can be coupled to a transducer surface to exhibit sensitive and reliable signals, which has enabled their application in clinical [18,19,49,50], environmental [51,52], agricultural and food diagnostics [53].

### 2.1. Imprinted Polymers: Preparation Strategies According to the Template’s Size

#### 2.1.1. Molecularly Imprinted Polymers

**Figure 1:**
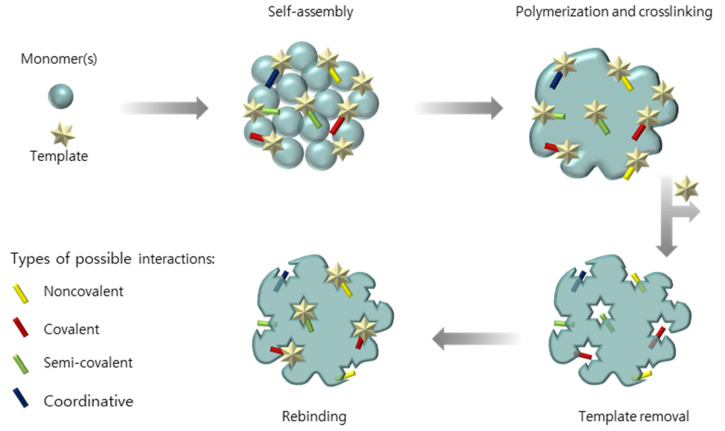
Synthetic steps to prepare MIPs.

MIPs are functional materials with specific recognition sites that, in a general view, are synthesized via the polymerization of functional monomers in the presence of a molecular template. The general procedure for synthesizing a MIP is illustrated in Figure 1**.** Firstly, the monomer and template are dissolved in an appropriate solvent (porogen). Due to intermolecular interactions that can be of covalent, noncovalent, semi-covalent or coordinative nature, the template and monomers self-assemble, forming a complex [15,17,54,55]. Subsequently, the monomers are polymerized and the material is cross-linked. Upon the removal of the template molecule, defined three-dimensional micro- or nano-cavities will remain in the polymer, able to recognize the original template. The supramolecular events of interaction (e.g., covalent or non-covalent) and shape complementarity resemble the natural mechanism of natural receptors toward their targets [55,56].

Even though self-assembly is the general principle by which MIPs are prepared, several experimental strategies leading to materials with different physicochemical characteristics can be followed. These preparation methods are briefly described in this section.

Bulk imprinting method is the oldest and most commonly reported strategy due to its simplicity. It typically employs free radical polymerization (FRP), which is carried out in solution with the aid of a thermal or UV initiator [57,58]. The use of FRP has the advantages of mild reaction conditions as well as suitability for various monomers (vinyl-containing molecules) and templates [42]. The solid monolith obtained after polymerization is subsequently ground and sieved to obtain MIP particles. Even though “bulk” preparation of MIPs is versatile and easily scalable, some disadvantages such as polymer loss and low binding kinetics as result of crushing the imprinted monolith have been identified [59,60].

Precipitation polymerization is a strategy that allows the synthesis of polymeric micro- or nano-spheres with a controlled morphology. As in bulk preparation of MIPs, FRP of the monomers is most commonly employed, with the experimental variant of using an excess of solvent in which the precursors (template and monomers) are soluble, but the forming polymer is not. As the reaction proceeds, polymer nuclei form, and, by growing, they segregate from the solution to form micro- or sub-micrometer-sized beads [61,62]. In contrast to the preparation of MIPs in the bulk, the polymer obtained does not require additional grinding steps.

Emulsion polymerization enables the formation of homogenous imprinted polymer particles. Similar to the two previously mentioned strategies, emulsion usually employs FRP. The reaction mixture, however, consists of a biphasic (typically aqueous and organic) system and a surfactant, which enables the formation of an emulsion and, thus, the growth of the polymer in the dispersed phase in a core–shell manner [63,64].

Solid-phase synthesis consists of the immobilization of the initiator or the template onto a solid support in order to carry out the polymerization directly from it. When immobilizing the template, it is possible to remove low affinity imprinted polymer as well as unreacted monomer after the synthesis [65]. On the other hand, when immobilizing the initiator, diverse controlled free radical polymerization techniques such as reversible addition fragmentation chain-transfer polymerization (RAFT) and atom transfer radical polymerization (ATRP) can be used with the advantages of obtaining more homogeneous polymer networks and thus higher target affinities in comparison to the traditional free radical polymerization [66,67,68].

Electro-polymerization consists of the oxidation of conjugated monomers and the deposition of a conductive polymer film onto a substrate. The synthesis system for these layers consists of a solution containing the monomer(s), the template, an electrolyte and three electrodes (working, reference and counter) [69]. Typically, the working electrode serves as deposition substrate for the material, and a wide variety of electrochemical techniques can be employed for the electrosynthesis (galvanostatic, potentiostatic and voltammetric). By adjusting the experimental conditions for electro-polymerization, it is possible to adjust the thickness, topology and morphology of the imprinted films [70,71,72].

Numerous reviews deal with the aspects of MIP synthesis and processing [17,61,73]. Although these biomimetic receptors have successfully proven their applicability for detecting low molecular weight compounds, larger molecules such as enzymes, proteins and cells turned out to be more challenging. The steps of template removal and rebinding are especially difficult since macromolecules cannot penetrate the small porous network in a straightforward manner [45,74,75,76]. These issues have been overcome by using different imprinting strategies, creating surface imprinted polymers (SIPs) [10,22,75,77].

#### 2.1.2. Surface Imprinted Polymers

**Figure 2:**
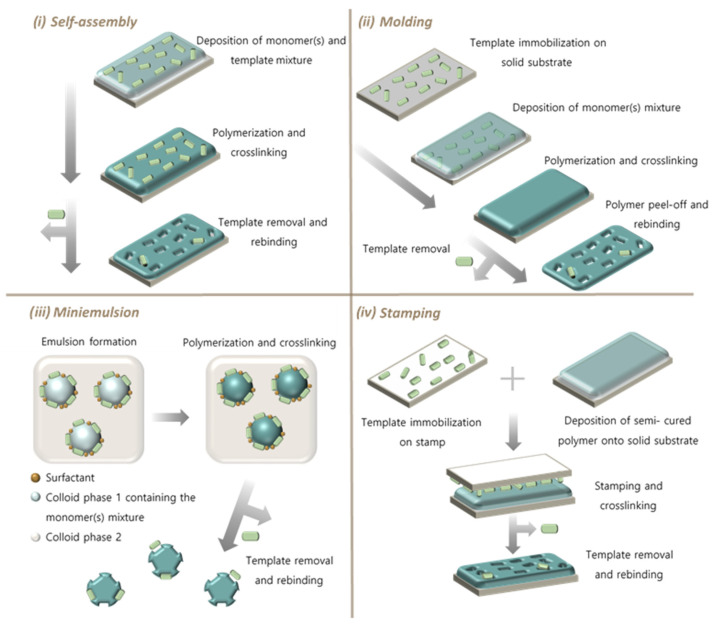
Synthetic steps to prepare SIPs: (**i**) self-assembly method; (**ii**) molding technique; (**iii**) miniemulsion polymerization; and (**iv**) stamping technique.

Synthesis of SIPs involve the fundamentals of MIP preparation; however, in SIPs the recognition sites are located at or near the surface of the forming polymeric material, facilitating the imprinting of macromolecular targets such as proteins, microorganisms or whole cells. Compared to traditional MIPs, SIPs exhibit higher binding capacity, faster mass transfer and binding kinetics [76]. Depending on how the polymer cavities are formed, SIPs can be classified as follows (Figure 2) [10]:

Self-assembly method: A polymerization mixture containing the functional monomers and template is prepared. The reaction is performed in a similar way as in traditional MIP synthesis, with the difference that, upon template removal, the only functional cavities formed are the ones situated on the surface of the material [78,79]. A variant for this method is the directed self-assembly of the monomer around the template driven by an applied physical force (electro-polymerization), which was introduced before for MIP preparation [80].

Molding technique: The template is immobilized on a solid substrate and the reaction mixture with monomers is added on top of it. The polymerization and crosslinking reactions are carried out, and, upon peeling off the polymer and removing the template, the cavities on the material’s surface are obtained [81,82].

Stamping or micro-contact printing technique: The functional monomers are semi-polymerized, and this material is applied onto a solid substrate. Separately, a monolayer of template particles is fixed on the surface of a stamp, which is subsequently pressed onto the semi-cured material. The cross-linking is completed in the presence of the stamp, such that, when this stamp is removed, the template has been transferred to the material, and, by taking it out, cavities are formed [50,83,84].

Mini-emulsion polymerization: The polymerization is performed in a colloidal system with the aid of a surfactant. Derived from this, the polymer grows in the physical form of small spheres, and, because the template particles arrange in the interphase of the colloid, the cavities are formed on the surface of the beads [85,86].

These methods have allowed MIPs/SIPs to be used for different purposes such as sample preparation, solid phase extraction and reaction catalysis, among others [87,88]. Moreover, these applications have been researched for application in the agricultural and food sectors due to the constant need of analytical techniques that aid to ensure food quality and safety, meeting the consumer’s requirements and avoiding FBD [89]. Although sensing based on biomimetic receptors has the potential of being a highly selective and useful tool in the detection of food safety hazards, reviews of IP in food science have focused mostly on extraction and separation applications [90,91,92,93]. Our aim is to fill this gap and highlight the multidisciplinary aspects involved in the application of imprinting technology in the whole value chain ranging from IP preparation to integrated sensor systems for the specific recognition and quantification of chemical and microbiological contaminants in food samples.

## 3. Chemical Food Hazards

A wide variety of chemical compounds can be the cause of FBDs. These substances can be naturally present in food, such as allergens, or can result from the production and processing steps. Agricultural chemicals (pesticides, fertilizers and veterinary drugs) are the most common chemical hazards from food production. Moreover, some compounds migrating from packaging, food additives and contaminants produced during storage also have the potential of causing health damage [94]. Due to the broad variety of food chemical hazards, this work focuses mainly on the imprinting strategies to recognize pesticides and drugs (agricultural chemicals) in food samples (Table 1 and Table 2), while an additional section briefly summarizes other chemical food contaminant categories (Table 3).

Agrochemicals are used worldwide. Pesticides, for example, have the purpose of preventing and destroying any unwanted species of plant or animal (e.g., weeds and insects) in foods in order to regulate their growth and prevent their deterioration [95]. Another class of chemicals frequently used in agriculture are pharmacologically active compounds, or drugs, which aim to ensure the health of animals involved in food production. Despite the beneficial role that pesticides and drugs have, inadequate use can cause short- or long-term adverse health effects. Pesticides (e.g., carbamates, organophosphates and pyrethroids) can have carcinogenic, cytotoxic and mutagenic effects [96,97,98]. On the other hand, administering drugs (antibiotics) to animals intended for human consumption can have negative effects such as the emergence, spreading and persistence of antimicrobial-resistant bacteria among humans [99]. Furthermore, the illicit use of these compounds in animals can turn into severe poisoning outbreaks, such as the ones originated in France (1990) and Spain (1992) due to the consumption of clenbuterol [100].

The detection of chemical residues in food is essential in the prevention of FBDs, and it often requires the accurate detection of trace concentration. Pesticides are usually quantified using gas chromatography–mass spectroscopy (GC-MS), while drugs are detected with liquid chromatography due to their more polar chemical nature [101]. However, analysis of agricultural chemistry in food safety remains a challenge due to the broad diversity in chemical compounds and their presence at low concentrations in complex matrices [102]. Due to their specificity, IPs have been developed to recognize pesticides and drugs in real food samples. Research efforts studying these receptors are aimed at the selection of the target chemical contaminant, selection of functional materials and choosing an imprinting strategy. The specific synthesis protocol of IPs has a huge influence on their selectivity, a fundamental aspect of technologies for food hazard detection [103].

### 3.1. Imprinting Technology for the Recognition of Chemical Food Hazards

Most chemical food hazards are low-molecular-weight compounds, which is the main reason MIPs have been extensively used for their detection. However, some surface imprinting techniques have also been adapted to create receptors for the recognition of these small analytes. This section aims to summarize how these diverse preparation strategies for IPs have been applied to the recognition of these analytes in real food samples.

#### 3.1.1. Pesticides

Abdel-Ghany et al. illustrated in their work how you could exploit traditional bulk preparation for the imprinting of dinotefuran insecticide using acrylamide and methacrylic acid as monomers with ethylene glycol dimethacrylate as cross-linker. Different compositions of these building blocks were tested. The obtained MIP powder was integrated into membranes with the aid of a plasticizer and then embodied into the sensing device, which was evaluated for the detection of the insecticide in cucumber and soil samples. Before the recognition experiments, samples were pre-treated by chopping and blending them, followed by sonication and centrifugation, steps that were performed with the aim of ensuring the extraction of the target. The recognition ability of the polymer was assessed by recovering the analyte from the real samples, obtaining average values in the range of 7.9–106.4%. Furthermore, the integration of the IP into the sensing device allowed the determination of dinotefuran with a limit of detection (LoD) of 0.35 µg L^−1^. Apart from these experiments, selectivity experiments against some related substances were performed [104].

Motaharian et al. presented a similar technique for recognition of diazinon. They used imprinted polymer beads, which were prepared by carrying out polymerization of the monomers (methacrylic acid and ethylene glycol dimethacrylate) in a biphasic system of chloroform and silicon oil. To incorporate the obtained spheres (<100 nm) into the sensor, the beads were mixed with graphite and paraffin oil as binder to prepare a carbon paste electrode. The synthetic receptor was employed to recognize the analyte in blended apple solutions, from which recovery values in the range of 92.5–100.8% were obtained. When combining the spherical receptors to the sensor, detection of the analyte was achieved with an LoD of 7.9 × 10^−10^ mol L^−1^ [105].

Li et al. demonstrated the use of electro-polymerized MIPs for the electrochemical detection of the pyrethroid pesticide cypermethrin. Dopamine and resorcinol were investigated in order to take advantage of the synergic affinity of the two different monomers. The imprinted polymer films were deposited by cyclic voltammetry on a glassy carbon electrode using phosphate buffer as electrolyte. The sensor obtained was a hybrid material composed of the IP and a support layer of doped zinc oxide, which was prepared on the surface of the electrode previously to the electrodeposition. The recognition element was then tested on mackerel and crayfish, samples from which the abdominal meat was homogenized, centrifuged and extracted in ethanol. The accuracy of the prepared sensor in these complex samples was verified with the observed recoveries from 96.2% to 100.4%. As for the application of the polymer film in the sensing platform, the LoD obtained was 6.7 × 10^−14^ M [106]. Over the years, a wide variety of similar MIP-based sensors have been developed for the detection of pesticides, which are summarized in Table 1.

**Table 1:** Summary of recent publications for the recognition of pesticides using IPs in food samples.

Analyte (s)	Template/Monomer(s)/Crosslinker	IP Preparation	Food Sample	LoD	Readout Technique	Ref.
Malathion	Malathion/Acrylamide/Bisacrylamide	Thermal: Bulk	Olive fruits, oils	0.06 pg/mL	Electrochemical	[107]
Cyromazine	Cyromazine/MAA/EGDMA	Thermal: Precipitation	Agricultural waste water, soil	2.6 × 10^−6^ M	Electrochemical	[108]
Dinotefuran	Dinotefuran/MAA/EGDMA	Thermal: Bulk	Cucumber, soil	0.35 µg/L	Electrochemical	[104]
Diazinon	Diazinon/MAA/EGDMA	Thermal: Suspension	Apple, well water	7.9 × 10^−10^ mol/L	Electrochemical	[105]
Dicloran	Dicloran/MAA/EGDMA	Thermal: Bulk	Water	4.8 × 10^−10^ mol/L	Electrochemical	[109]
Lindane	Lindane/MAA/EGDMA	Thermal: From MWCN surface	Orange, grape, tomato, cabbage	1.0 × 10^−10^ M	Electrochemical	[110]
Carbaryl	Carbaryl/MAA/EGDMA	Thermal: From QD surface	Chinese cabbage, rice	1.47 × 10^−7^ mol/L	Optical	[111]
Metolcarb	Metolcarb/MAA/EGDMA	Thermal: Bulk	Apple juice, pear, cabbage	2.309 µg/L	Acoustic wave	[112]
Cyanazine	Cyanazine/Acrylamide/EGDMA	Thermal: Bulk	Onion, tomato, lettuce	3.2 nM	Electrochemical	[113]
Phosalone	Phosalone/APTES/TEOS	Thermal: Sol-gel	Cucumber, orange, wheat, water, soil	0.078 nM	Electrochemical	[114]
Trichlorfon	Trichlorfon/Vinylidene difluoride	Pre-synthesized polymer	Lettuce	4.63 ppb	Acoustic wave	[115]
2,4-dichlorophenol	2,4-dichlorophenol/3,4-EDOT	Electrochemical: Deposition	Water	0.07 nM	Electrochemical	[116]
Carbendazim	Carbendazim/*o*-phenylenediamine	Electrochemical: Deposition	Diverse fruits and vegetables	6.7 × 10^−13^ M	Electrochemical	[117]
Cypermethrin	Cypermethrin/Dopamine, resorcinol	Electrochemical: Deposition	Soil, mackerel, crayfish, water	6.7 × 10^−14^ M	Electrochemical	[106]
Methyl-parathion	Methyl-parathion/Resorcinol, quercetin	Electrochemical: Deposition	Fruit surfaces	3.4 × 10^−10^ mol/L	Electrochemical	[118]

MAA, Methacrylic acid; EGDMA, ethylene glycol dimethacrylate; APTES, (3-aminopropyl) triethoxysilane; TEOS, Tetraethyl orthosilicate; EDOT, Ethylenedioxythiophene; TRIM, Trimethylopropane trimethacrylate; MAGA, Methacryloylamidoglutamic acid; HEMA, Hydroxyethyl methacrylate; APBA, 3-aminophenylboronic acid, QD, Quantum dots; MWCN, Multi-walled carbon nanotubes; MNP, Magnetic nanoparticles.

As pesticides are low-molecular weight compounds, the literature is focused on the creation of MIPs rather than SIPs. Bulk polymerization has been demonstrated to be suseful and has even led to the development of sensitive platforms that can compete in LoD with sensors using MIPs created via more advanced routes. This offers the benefit of being able to make proof-of-concept platforms in a fast manner. Suspension and precipitation polymerization platforms are in general a bit more sensitive but are also more suitable for upscaling due to their increased homogeneity. In terms of scalability, electro-polymerized MIPs offer a very interesting approach in constructing MIP-based sensors but, as evidenced by the results in Table 1, are often only combined with electrochemical detection platforms and require a long process of fine-tuning.

#### 3.1.2. Drugs

In addition to pesticides and insecticides, a wide range of MIP sensors that focus on the detection of drugs in food samples has been developed in the past few decades. In 2013, Chen et al. made use of core–shell imprinted particles that were prepared for the detection of metronidazole antibiotic. The IP was synthesized using (3-aminopropyl)triethoxysilane and tetraethyl orthosilicate as monomers. The polymerization reaction was carried on the surface of silica coated Fe_3_O_4_ nanoparticles, which were employed as solid support due to their chemical stability and magnetic properties. The obtained IP particles were drop casted onto a magnetic glassy carbon electrode. The receptor was tested for the recognition of the antibiotic in milk and honey samples, which were prepared by homogenization, centrifugation and filtration before the rebinding experiments. The performance of the synthetic receptor in these samples was assessed with the recovery percentages, which were 93.5–102.2%. The magnetic particles were further tested in a sensing platform, which attained a LoD of 1.6 × 10^−8^ M [119].

Wei et al. demonstrated the use of electro-polymerized MIPs for the detection of the antibacterial agent sulfadimine in 2019. Electrodeposition of the conjugated monomer polypyrrole was performed on the surface of NiCo_2_O_4_ nanoneedle arrays in order to construct a highly selective MIP-sensor. The nanoneedles were selected for their use with IP due to their high specific surface area, which enables them to enhance the performance of detection. These arrays were synthesized and decorated onto a graphene electrode, which was subsequently used as working electrode in the electropolymerization via cyclic voltammetry using tetrabutylammonium perchlorate as supporting electrolyte. The fabricated electrode was employed for the detection of this drug in milk. The food samples were pre-treated to remove protein and dissolve organic substances with acetonitrile and trichloroacetic acid. Recovery values from these samples were of 92.3–102.2%. Additionally, the application of the nanoneedle array/pyrrole in a sensing device led to a LoD of 0.169 ng mL^−1^ [120]. Other examples of MIP-based drug detection in food samples are summarized in Table 2.

**Table 2:** Summary of recent publications for the recognition of drugs using IPs in food samples.

Analyte (s)	Template/Monomer(s)/Crosslinker	IP Preparation	Food Sample	LoD	Readout Technique	Ref.
Amantadine and rimantadine	Amantadine/MAA/EGDMA	Thermal: Bulk	Chicken, pork	1.0 pg/mL	Optical	[121]
Chloramphenicol	Chloramphenicol/MAA/EGDMA	Thermal: Bulk	Milk	2.0 × 10^−9^ M	Electrochemical	[122]
Chloramphenicol	Chloramphenicol/MAA/EGDMA	Thermal: Bulk	Milk	10 µM	Electrochemical	[123]
Clenbuterol	Clenbuterol/MAA/EGDMA	Thermal: Bulk	Bovine liver	0.2 nM	Electrochemical	[124]
Sulfonamides	Sulfabenz/MAA/EGDMA	Thermal: Bulk	Chicken, pork	1–12 pg/mL	Optical	[125]
Kanamycin	Kanamycin/MAA/EGDMA	Thermal: From MWCN surface	Chicken, pig, milk	2.3 × 10^−11^ mol/L	Electrochemical	[126]
Sulfaguanidine	Sulfaguanidine/MAA/EGDMA	Thermal: Bulk	Fish	2.8 × 10^−10^ mol/L	Optical	[127]
Quinolones	Enrofloxacin/MAA/EGDMA	Photo: Bulk	Fish	4.06 × 10^−7^ µmol/L	Optical	[128]
Benzimidazoles	Mebendazole, fuberidazole/MAA/EGDMA	Thermal: Bulk	Mutton, beef	21 pg/mL	Optical	[129]
Chloramphenicol	Chloramphenicol/MAA/TRIM	Thermal: Bulk	Prawns	7.8 × 10^−8^ µM/mL	Acoustic wave	[130]
Oxytocin	Oxytocin/MAGA,2-HEMA/ EGDMA	Thermal: Bulk	Milk	0.003 ng/mL	Optical	[131]
Estradiol	Estradiol/Aniline	Thermal: Bulk	Milk powder	2.76 nmol/L	Electrochemical	[132]
Diethylstilbestrol	Diethylstilbestrol/APBA	Thermal: From MNP surface	Milk	2.5 × 10^−10^ mol/L	Electrochemical	[133]
Metronidazole	Metronidazole/APTES/TEOS	Thermal: Bulk	Milk, honey	1.6 × 10^−8^ M	Electrochemical	[119]
Vitamin K_3_	Vitamin K_3_/3,4-EDOT	Electrochemical: Deposition	Poultry drug powder	3.1 × 10^−4^ µM	Electrochemical	[134]
Tobramycin	Tobramycin/Pyrrole	Electrochemical: Deposition	Egg, milk	1.4 × 10^−10^ M	Electrochemical	[135]
Oxfendazole	Oxfendazole/Pyrrole	Electrochemical: Deposition	Milk	10 µg/Kg	Electrochemical	[136]
Sulfadimidine	Sulfadimidine/Pyrrole	Electrochemical: Deposition	Milk	0.169 ng/mL	Electrochemical	[120]
Streptomycin	Streptomycin/*o*-phenylenediamine	Thermal: Suspension	Milk, honey	10 pg/mL	Electrochemical	[137]

MAA, Methacrylic acid; EGDMA, ethylene glycol dimethacrylate; APTES, (3-aminopropyl) triethoxysilane; TEOS, Tetraethyl orthosilicate; EDOT, Ethylenedioxythiophene; TRIM, Trimethylopropane trimethacrylate; MAGA, Methacryloylamidoglutamic acid; HEMA, Hydroxyethyl methacrylate; APBA, 3-aminophenylboronic acid; MWCN, Multi-walled carbon nanotubes; MNP, Magnetic nanoparticles.

Looking at the summary in Table 2, the same conclusions that apply for pesticides can also be drawn for MIP-based drug detection in food products. Bulk imprinted MIPs offer a fast track solution to constructing a first prototype sensor that is surprisingly sensitive and can compete with more advanced systems incorporating for instance MWCNs into the synthetic receptor layer. This does boost the sensitivity of an electrochemical sensor but not by multiple orders of magnitude. Electro-deposition again proves to be an excellent match for electrochemical detection of drug molecules offering fast synthesis, immediate deposition and therefore scalability.

#### 3.1.3. Other Chemical Contaminants

In addition to pesticides, insecticides and drugs, other chemical agents such as allergens can also pose a health risk (Table 3). In 2018, Ashley and coworkers demonstrated the use of imprinted nanoparticles for the detection of casein, a naturally occurring allergen in dairy products. Casein molecules were immobilized on glass beads, and solid-phase polymerization was used for the preparation of the imprinted receptors on these solid substrates. The monomers selected for the receptor were N-isopropylacrylamide, *N*-*tert*-butylacrylamide, acrylamide, *N*-(3-aminopropyl)-methacrylamide and N,N’-methylenebis (acrylamide), and the obtained particles exhibited physical dimensions from 235 to 457 nm. The nanoparticles were tested on washed samples from cleaning in place systems used in food processing equipment, from which recoveries ranged 87–120%. When employing the recognition ability of the nano-MIPs in a sensing device, the authors reported a limit of detection of 0.127 ppm. Remarkably, this LoD is superior to the sensitivity typically encountered in commercial ELISA kits [138].

**Table 3:** Summary of recent publications for the recognition of other chemical contaminants using IPs in food samples.

Category	Analyte (s)	Template/Monomer(s)/Crosslinker	IP Preparation	Food Sample	LoD	Readout Technique	Ref.
Naturally occurring	α-Casein	α-Casein/NIPAm, TBA, AA, APM/BIS	Thermal: From glass beads	CIP from dairy ice cream	0.127 ppm	Optical	[138]
Additives	Sudan dyes	PN/MAA/EGDMA	Photo: Solution	Egg yolk	1 pg/mL	Optical	[139]
Additives	Sudan I	Sudan I/2-vinylpyridine/EGDMA	Thermal: From attapulguite NC	Tomato sauce, sausage, water	0.01 ng/mL	Optical	[140]
Naturally occurring	Histamine	Histamine/MAA/EGDMA	Thermal: from SPR chip	Carp	25 µg/L	Optical	[141]
Naturally occurring	Histamine	Histamine/MPTES/TEOS	Sol-gel	Fish	7.49 × 10^−4^ mg/kg	Acoustic wave	[142]
Production, packaging	Bisphenol A	Bisphenol A/TEOS/APTES	Sol-gel	Water, milk	1.46 × 10^−11^ M	Optical	[143]
Production	Acrylamide	Bisphenol A/APTES/TEOS	Sol-gel	Potato chips	0.028 µg/mL	Electrochemical	[144]
Production	Acrylamide	Propionamide/HEA/EGDMA	Thermal: From GO electrode	Potato chips	0.01 µg/mL	Optical	[145]
Production	Melamine	Melamine/MAA/EGDMA	Photo: From Au electrode	Milk	3.1 × 10^−10^ mol/L	Electrochemical	[146]
Production	Melamine	Melamine/*para*-ABA	Electrochemical: Deposition	Milk	0.36 µM	Electrochemical	[147]
Production	Melamine	Melamine/Pyrrole	Electrochemical: Deposition	Milk	0.83 nM	Electrochemical	[148]

CIP, Clean in place; NIPAm, N-isopropylacrylamide; TBA, N-tert-butylacrylamide; AA, Acrylic acid; APM, N-(3-aminopropyl)-methacrylamide; BIS, N,N’-methylenebis (acrylamide); MAA, Methacrylic acid; EGDMA, ethylene glycol dimethacrylate; 3-MCPD, 3-chloro-1,2-propanediol; ABA, Aminobenzoic acid; APTES, (3-aminopropyl) triethoxysilane; TEOS, Tetraethyl orthosilicate; HEA, Hydroxy ethyl acrylate; PN, 1-(2-pyridinylazo)-2-naphthol;MPTES, 3-mercaptopropyltriethoxysilane; NC, Nanofibrillar clay; SPR, Surface plasmon resonance; GO, Graphite oxide.

MIP-based sensors for other chemical contaminants such as endocrine disruptors, toxins or chemicals typically employ more advanced imprinting technologies. This might be explained by the fact that bulk imprinting has already been done on these types of molecules in the more distant past, demonstrating the concept but also the limitations. The sensors in Table 3 offer a lot of benefits in term of mass-scale production as both electro-deposition and direct grafting onto gold or graphene electrodes allow for upscaling of production, which might be the biggest challenge when commercializing imprinting technology. Direct grafting onto electrodes offers the benefit of extending the potential transduction mechanisms used from electrochemical to optical or gravimetric techniques.

## 4. Biological Food Hazards

Diarrheal and gastroenteritis diseases account for the majority of the yearly estimated 600 million cases of foodborne illnesses around the world [149,150]. The mentioned disorders are primarily caused by biological contaminants present in food, which can be organisms such as parasites, microorganisms such as fungi and bacteria or viruses [151].

Among biological food hazards, bacteria are the most recurrent cause of disease [152], with *Salmonella* species, *Campylobacter jejuni* and *Escherichia coli* some of the most commonly involved foodborne pathogens [3,153]. The detection of these microorganisms is typically carried out via culture and colony counting, polymerase chain reaction (based on the amplification and quantification of the microorganism’s DNA) and immunology-based methods [154,155,156,157]. The disadvantages of these strategies are that colony counting can be highly time-consuming and error-prone, while polymerase chain reaction methods usually require expensive equipment and reactants as well as specialized technical skills for performing them [158,159]. Finally, immunology techniques may require from hours to days to provide a result [160].

Certain types of bacteria are able to produce toxins, molecules that are also secondary metabolites of some fungi and represent a food safety hazard [161,162,163]. Their adverse health effects can vary; while some toxins such as staphylococcal can cause enteric illness, aflatoxins (produced by fungi) are known for being carcinogenic [164,165]. The detection of these agents is thus crucial for preventing foodborne outbreaks. Typically, these biological derived contaminants are detected via protocols that consists of time consuming extraction from the food matrix followed by pre-concentration steps and liquid or gas chromatography for their quantification [166].

Foodborne viruses are capable of infecting intestinal cells and are shed in the stool [167]. Noroviruses and Hepatitis A are the most common cause of viral foodborne disease, causing gastroenteritis and hepatitis, respectively [168]. Typically, they are detected by the scanning of a stool suspension under an electron microscope. This protocol, however, is insensitive and labor-intensive [169]. Immunoassays are available for the detection of some viruses [170,171], as well as transcriptase-polymerase chain reaction [172,173]. Nonetheless, the drawbacks of these techniques have been already discussed.

New technologies have been developed to overcome the disadvantages of analytical methods for biological food hazards detection. Imprinted polymers have been extensively researched for the extraction of toxins, which has also led to the application of these synthetic receptors in sensors [174,175,176,177]. Furthermore, bacteria detection using IPs in sensing devices has also been exploited [28,30,178,179,180]. The use of synthetic receptors as recognition element in food safety sensing exhibits the potential of becoming a fast, sensitive and cost-effective technology in contrast to the traditional analytical methods [181,182,183].

### 4.1. Imprinting Technology for the Recognition of Biological Food Hazards

As mentioned above, the affinity of imprinted polymers relies on a combination of functionality and geometry between the targeted analyte and the receptor. In the recognition of biological targets, geometry plays a crucial role in the selection of the material to be employed for IP preparation. While some biological analytes allow the use of classical molecularly imprinting strategies due to their small sizes and low molecular weights (e.g., toxins and viruses), larger ones represent a challenge. Bacteria, for instance, are microorganisms with dimensions up to several microns depending on the taxonomic groups [184]. To ensure these template’s removal and rebinding, the synthesis of the receptor should allow the surface imprinting of the material [185].

This section is subdivided into the biological food hazards categories that have been detected using IPs. A summary of these agents, as well as the exemplification of the imprinting technologies employed for their recognition in food samples, is discussed.

#### 4.1.1. Toxins

The most straightforward method of detecting microbial contamination in food is the detection of the toxins they produce as, these are often relatively small molecules which makes it possible to detect them with MIPs (Table 4). Sergeyeva et al. created functional polymeric membranes that functioned as synthetic receptors for the mycotoxin Aflatoxin B1. Ethyl-2-oxocyclopentanecarboxylate was employed as dummy template in the preparation of the imprinted materials, which were obtained via the photo-polymerization of diverse monomers between two glass slides. From the tested building blocks, 2-acrylamido-2-methyl-1-propanosulfonic acid and acrylamide exhibited optimal performance for the recognition of the toxin. Recoveries of the analyte from the real samples wheat and maize flower ranged 87–96%. The prepared membranes were further assessed as recognition element in a sensing device, which attained an LoD of 20 ng/mL [186].

**Figure 3:**
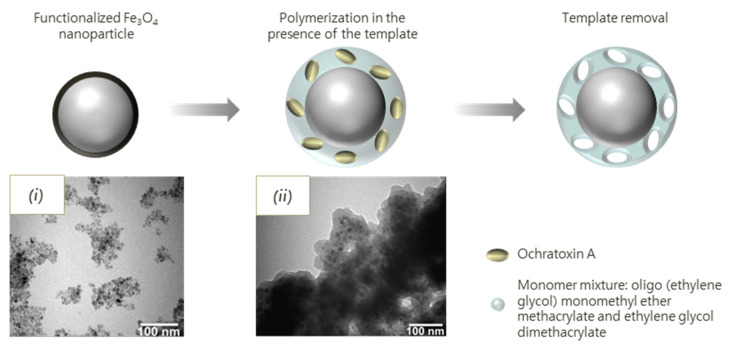
Schematic representation of synthesis of imprinted polymer on magnetic nanoparticles surface. TEM images of: (**i**) functionalized nanoparticles; and (**ii**) imprinted polymer magnetic nanoparticles. Reprinted from [187]. Copyright (2016), with permission from Elsevier.

Turan et al. made use of imprinted magnetic particles for the detection of ochratoxin A mycotoxin. For the preparation of the IPs, Fe_3_O_4_ nanoparticles were synthesized and surface-modified in order to act as polymerization initiators. Oligo(ethylene glycol) monomethyl ether methacrylate and ethylene glycol dimethacrylate were then polymerized from the particles in the presence of the template (Figure 3). The receptor was tested on spiked grape juice samples, observing a recovery of 97.1–97.5%. Additionally, sensing of the toxin was reported to be possible from 0.374 µg mL^−1^. Selectivity of the IP was tested using aflatoxin and vomitoxin as competitive mycotoxins, for which they observed lower adsorption capacities in comparison to the targeted ochratoxin A [187].

**Table 4:** Summary of recent publications for the recognition of toxins using IPs in food samples.

Analyte (s)	Template/Monomer(s)/Crosslinker	IP Preparation	Food Sample	LoD	Readout Technique	Ref.
*Staphylococcal* enterotoxins A,B	*S.* enterotoxin B/APTES, OTES/TEOS	Sol-gel	Milk	7.97 ng/mL	Acoustic wave	[188,189]
Patulin	2-oxin/APTES/TEOS	Sol-gel	Apple and pear juice, haw flakes	3.1 × 10^−3^ µg/mL	Acoustic wave	[190]
Patulin	2-oxindole/ρ-aminothiophenol	Electrochemical: Deposition	Apple juice	7.57 × 10^−13^ mol/L	Electrochemical	[191]
Patulin	6-Hydroxynicotinic acid/APTES/TEOS	Sol-gel	Apple juice	0.32 µmol/L	Optical	[192]
Ochratoxin A	Ochratoxin A/Pyrrole	Electrochemical: Deposition	Beer and wine	0.0041 µM	Electrochemical	[193]
Ochratoxin A	Ochratoxin A/OEGMA/EGDMA	Thermal: From MNP surface	Grape juice	0.374 µg/mL	UV-Vis	[187]
Zearalenone	Zearalenone/Pyrrole	Electrochemical: Deposition	Corn	0.3 ng^−1^	Optical	[194]
Zearalenone	CDHB/MAA/EGDMA	Thermal: Solution	Corn, rice and wheat flour	0.002 µmol/L	Optical	[195]
Deoxynivalenol	Deoxynivalenol/*o*-phenylenediamine	Electrochemical: Deposition	Corn	0.3 ng/mL	Electrochemical	[196]
Aflatoxin B1	ethyl-2-OPC/AA, allylamine, DEAEM, MBAA, AMPSA	Photo: Solution, semi-interpenetrating networks	Maize flour	20 ng/mL	Optical	[186]

APTES, (3-aminopropyl) triethoxysilane; TEOS, Tetraethyl orthosilicate; OTES, Triethoxy (octyl) silane; OEGMA, oligo (ethylene glycol) monomethyl ether methacrylate; EGDMA, ethylene glycol dimethacrylate; OPC, oxocyclopentanecarboxylate; DEAEM, 2-(diethylamino) ethyl methacrylate; AMPSA, 2-acrylamido-2-methyl-1-propansulfonic acid; MBAA, N,N’-methylenebisacrylamide.

The results in Table 4 are in line with the findings described in Section 3, which makes sense as toxins are typically low-molecular weight molecules similar to drugs, pesticides or hormone disruptors. Electro-deposition is again demonstrated to be a very interesting and well-researched approach due to its scalability and its combination with electrochemical transducers leading to sensitive sensor platforms.

#### 4.1.2. Bacteria

**Figure 4:**
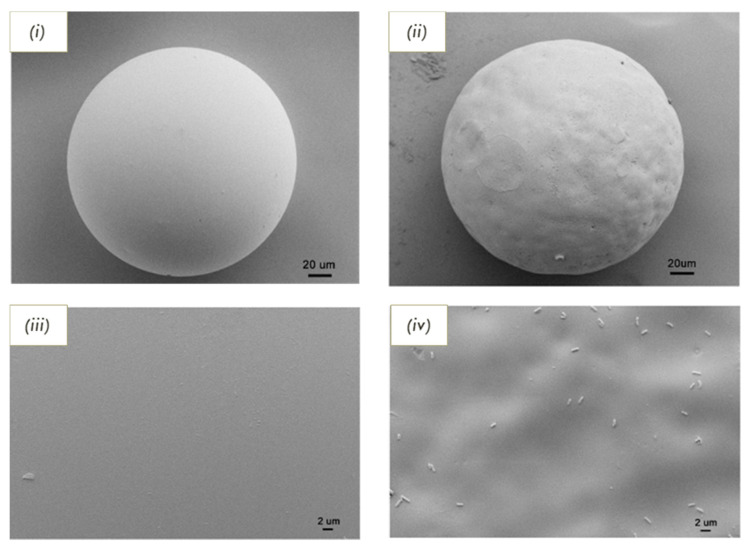
Scanning electron microcopy (SEM) images of (**i**) non-Imprinted particle and (**ii**) imprinted particle; and corresponding magnified images of (**iii**) non-imprinted and (**iv**) imprinted beads. Figure adapted from [197] with permission under open-access copyright agreement.

The detection of whole bacterial cells was demonstrated on several occasions over the past few years. In 2019, Zhao et al. used a Pickering emulsion polymerization approach for imprinting polymers with *Listeria monocytogenes*. The reaction mixture consisted of an oil–water system, in which the aqueous phase contained a bacteria–chitosan network, prepared with acryloyl-functionalized chitosan and quantum dots (CdTe). On the other hand, the oil phase contained the monomers (trimethylolpropane trimetharylate, N,N-dimethylaniline and divinylbenzene) and the initiator. Solid polymer beads with imprints on the surface were obtained, as can be seen in Figure 4. To assess the adsorption of the particles, binding experiments were performed, from which the adsorption capacities of the IP (355.6 CFU mg^−1^) exhibited a 4.5-fold increase in comparison to the non-imprinted polymers. Regarding the limit of detection, the sensor achieved a performance of 1 × 10^3^ colony forming units per milliliter. Furthermore, selectivity against *S. aureus* was visually confirmed. Application to real samples was carried out by inoculating milk and pork with the bacteria, homogenizing with the IPs and sedimenting for 3 min [197].

Cornelis et al. took a different approach in the construction of their bacterial sensor. They made synthetic *E. coli* receptors by micro-contact imprinting. The monomers employed were bisphenol A, phloroglucinol and 4, 4′-diisocyanatodiphenylmethane, which were partially polymerized before spin coating solid substrates. The surface imprinting of these polymers was performed with polydimethylsiloxane bacterial stamps. After full curing of the films in the presence of the template, functionalized polyurethane films were obtained. The films were incorporated into a sensing platform, which was able of detecting 1 × 10^2^ CFU mL^−1^. Cross-sensitivity was assessed with four coliform species of the Enterobacteriaceae family and the receptor was further tested with non-cleared spiked apple juice samples [198].

**Figure 5:**
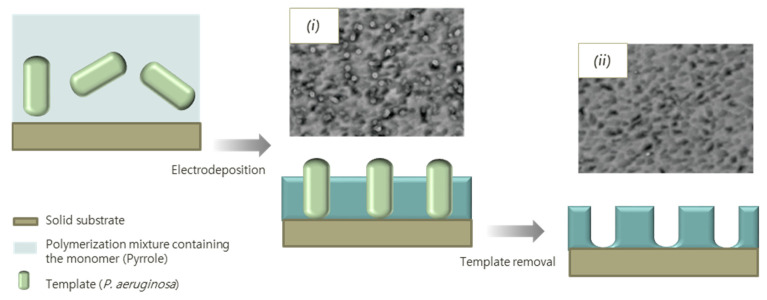
Schematic representation of polypyrrole imprinting with bacteria. SEM images of: (**i**) imprinted polypyrrole; and (**ii**) polypyrrole film with bacteria cavities. Reprinted (adapted) with permission from [80]. Copyright (2013) American Chemical Society.

Tokonami et al. *achieved* recognition of *Pseudomonas aeruginosa* by preparing an IP via the electropolymerization of pyrrole (Figure 5). The constant potential electrodeposition of the polymer was performed in phosphate buffer using a quartz crystal microbalance electrode as deposition substrate, same that was employed as sensor component for measurements of the bacteria in apple juice. The receptors, besides recognizing *P. aeruginosa* with a LoD of 1 × 10^3^ CFU mL^−1^, were able to discriminate the analyte against *E. Coli* and other bacterial species [80].

A summary of publications reporting on bacterial recognition employing IPs can be found in Table 5. As bacteria are whole-cell microorganisms, they require a different approach as compared to the sensor systems described above for small molecules. Although it is possible to use emulsion polymerization in MIP-like fashion [197], most platforms are based on SIP-based detection. Micro-contact imprinting offers the possibility of creating proof-of-concept platforms with surprisingly low detection limits, but upscaling would require a highly advanced roll-to-roll coating device. Electro-deposition in this sense is a valuable alternative leading to ultra-sensitive detection of bacteria in solution but requires voltammetry parameter optimization and is mainly interesting for electrochemical detection, while micro-contact imprinting is more broadly combinable with other readout technologies.

#### 4.1.3. Viruses

**Figure 6:**
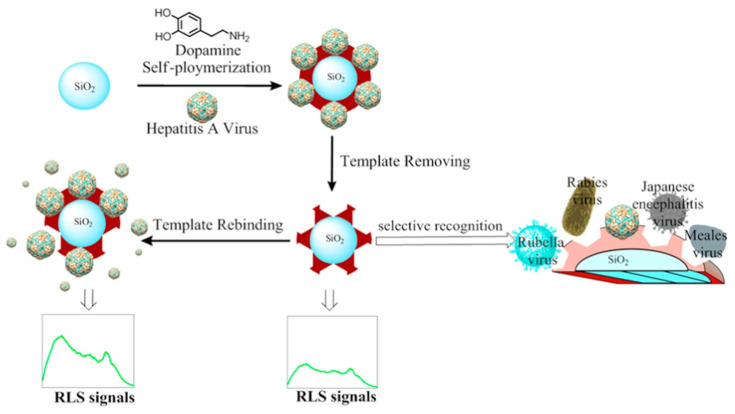
Schematic representation of the preparation of Hepatitis A-imprinted poly(dopamine). Reprinted from [201]. Copyright (2017), with permission from Elsevier.

To our knowledge, Yang et al. are the only group to report on the recognition of a virus in terms of food safety. In this research, silica nanoparticles were synthesized and used for molding imprinting of hepatitis A. Self-polymerization of the monomer (dopamine) was carried out in Tris buffer (Figure 6). The obtained imprinted particles were tested in human serum, from which they obtained recoveries in the range of 94–96%. The spherical IP proved to be able of sensing the analyte with a LoD of 8.6 pmol per liter. Moreover, selective recognition against other viruses (rubella, rabies and measles) viruses was performed [201].

## 5. The Role of Transducers in Designing a Sensor for Application in Food Safety

**Figure 7:**
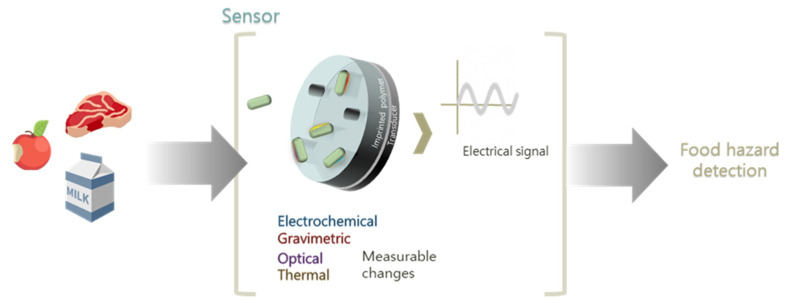
Imprinted polymers coupling to transducers for food safety application.

In the previous sections, we mainly focus on the different potential contaminants and how different imprinting strategies can be exploited to create sensitive receptors for both sensing and separation/purification purposes. When constructing integrated sensor devices, however, the transducer principle used is equally important to the receptor design as the sensitivity of a sensor depends on both the recognition element and the transducer. The transducer possesses the key role of converting the recognition event into a measurable signal, typically proportional to the number of interactions between the analyte and the receptor (Figure 7) [37,202]. In this section, we describe the most representative readout techniques that have been combined with IPs in the detection of chemical and microbiological food contaminants.

### 5.1. Electrochemical Detection

Sensors based on electrochemical transducers are the most frequently reported in biosensing applications due to their relatively high sensitivity and selectivity at a low cost [203,204]. The possibility of miniaturization and the suitability that these platforms possess for being used in complex matrices are two additional key advantages for food analysis [205]. In this regard, IPs have been coupled with electrochemical transducers to sense food safety hazards. Upon the binding of the specific targets to the receptor, electrical changes are observed. These changes can be amperometric/voltammetric (in currents) or potentiometric (in potentials or accumulated charge). Additionally, impedimetric changes (in resistance or capacity) have also been researched [206]. Since electrochemical transducers rely on measurable electrical changes, the electron transfer ability between the recognition sites of the receptor and the surface of the electrode employed determines the sensitivity of the sensor [207]. Therefore, conductive materials such as conjugated polymers or conductive additives as nanoparticles (carbon, gold, etc.) are typical components of these platforms.

**Figure 8:**
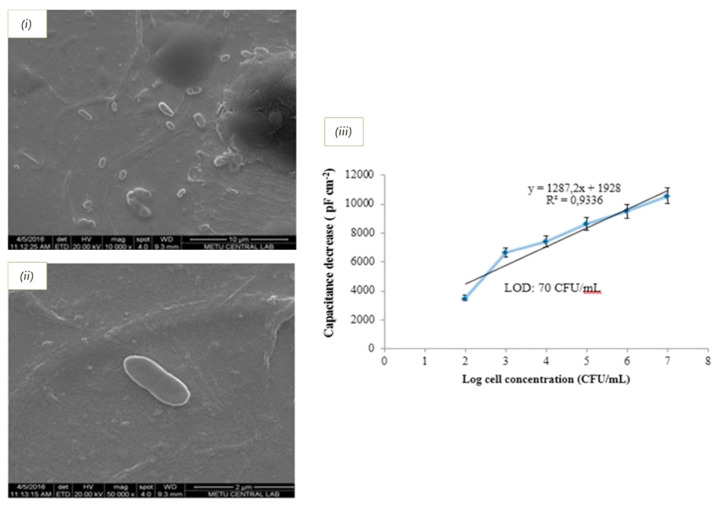
Electrochemical detection of Escherichia coli: (**i**,**ii**) SEM images of bacteria imprinted electrodes; and (**iii**) calibration curve obtained from the capacitive response of the electrode towards the analyte. Reprinted from [209]. Copyright (2017), with permission from Elsevier.

For example, Bougrini et al. used voltammetry to quantify tetracycline, using an imprinted layer composite of poly(thioaniline) and gold nanoparticles onto a gold electrode for the measurements. The sensor attained a limit of detection of 0.22 fM and was successfully tested on honey samples, proving the suitability of the sensor in complex food matrices [208]. Apart of high sensitivity, rapid detection is a desirable characteristic in sensors for food safety, where toxin accumulation during time poses a risk. This challenge was addressed by Idil et al. for the real time detection of E. *coli,* using a capacitive transducer coupled to a vinyl based imprinted polymer onto a gold electrode. This sensor yielded a detection limit of 70 CFU/mL and was tested on apple juice samples (Figure 8) [209]. Other related publications that cover electrochemical platforms coupled to imprinted polymers are summarized in Table 6.

### 5.2. Optical Detection

The core process of these sensors is the conversion of optical properties (e.g., photons) into an electronic signal, related to a receptor–target binding event. Reports of IP sensors exploit optical properties such as refractive index, optical absorbance or fluorescence [215]. Other well-known examples of optical biosensing platforms include Surface Plasmon Resonance (SPR) and Surface Enhanced Raman Scattering (SERS). In terms of MIPs and food hazards detection, fluorescence and SPR are the most investigated readout platforms (Table 7).

Fluorescence is often used due to its simplicity and high sensitivity. Sensors based on fluorescence, however, present the drawback of requiring a label when the analyte does not possess intrinsic optical properties (e.g., absorption or photoluminescence). One of the most representative fluorescent labels are quantum dots (QD), which can be incorporated into the synthetic receptor to enable the use of this readout method [36,216]. An example within food hazard detection is the sensor employed by Liu et al. for methamidophos, a pesticide. The imprinted polymeric receptor is based on methacrylic acid, ethylene glycol methacrylate and QDs as label. The obtained fluorescent probe showed a limit of detection of 0.092 μM, and the setup was tested on real food matrices such as apples and pears [217].

Under specific incident light illumination, SPR measures changes in the refractive index of a medium near a metal surface (e.g., Au or Ag). This optical phenomenon is exploited for biosensing, where receptors are immobilized on the surface. Upon binding to their specific targets, a change in mass will be observed and, thus, a change in refractive index that can be analyzed in real time [218,219]. Zhang et al. demonstrated the use of an SPR biomimetic sensor for the detection for the antibiotic kanamycin in 2018. They obtained a limit of detection of 0.043 μM in complex food samples, e.g. milk and honey [220].

**Table 7:** Imprinted polymer-optical sensors for food safety.

Analyte	Sensor Composition IP/Electrode	Food Sample	Limit of Detection	Response Time	Ref.
Kanamycin	Poly 4-vinylphenylboronic acid-*co*-PEGDA)	Milk, honey	0.0433 μM 0.0120 μM	-	[220]
Methamidophos	Poly (MAA-*co*-EGDMA),QD	Apple, pear, kidney bean, leek, cucumber	0.0916 μM	3 h	[217]
*Escherichia coli*	Poly(HEMA-*co*-EGDMA-*co*-MAH)	Apple juice	1.54 × 10^6^ CFU/mL	113 s	[200]

PEGDA, Poly (ethylene glycol) diacrylate; MAA, Methacrylic acid; EGDMA, Ethylene glycol dimethacrylate; QD, Quantum dots; MAH, N-methacryloyl-L-histidine methylester; HEMA, 2-Hydroxyethyl methacrylate.

### 5.3. Acoustic Wave Transducers

As these sensors detect small changes in mass, they are also known as mass-sensitive sensors. They typically comprise a piezoelectric crystal, onto which the receptors (in this case, imprinted polymers) are immobilized. The crystal will vibrate at a certain frequency under the influence of an electrical field, achieving resonance at a very high frequency. When the target binds to the receptor, it will produce a measurable change in the crystal’s vibration frequency. This change correlates with the added mass on the crystal surface [221]. Some of its advantages lie in the possibility to perform several assay formats as well as on-line analysis of receptor–target interactions [219]. Nevertheless, the relatively long incubation times required as well as the number of washing and drying steps can be drawbacks in terms of application for food safety.

**Figure 9:**
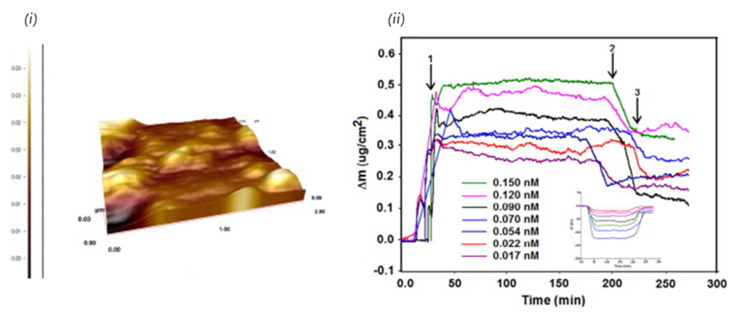
QCM detection of tobramycin using a molecularly imprinted polymer: (**i**) AFM image of MIP film. (**ii**) The effect of concentration on QCM response of target: (1) adsorption; (2) desorption; and (3) regeneration. Reprinted from [222]. Copyright (2014), with permission from Elsevier.

There are two major types of mass-based biosensors: Bulk Acoustic Wave (BAW) and Surface Acoustic Wave (SAW) devices. BWV is also known as Quartz Crystal Microbalance (QCM), and, as shown in Table 8, it is the piezoelectric transducer most exploited for the detection of food safety hazards of both chemical and microbiological nature in real samples. Some of these sensor platforms have been proven to be very sensitive and highly interesting for application in food safety screening (Table 8). Yola et al., for example, used a QCM-based biomimetic sensor for the real-time detection of the antibiotic tobramycin in chicken egg white and milk in concentrations in the low picomolar concentration range (Figure 9) [222].

### 5.4. Thermometric: Heat Transfer Method

A relatively novel class of transducer principles are thermal detection techniques. Traditionally, thermal sensing principles are usually based on calorimetry, but, in recent years, the Heat Transfer Method (HTM) has emerged as an interesting biosensing technique [10]. HTM monitors the thermal transport across a solid-liquid interface where the IP receptors are immobilized. Moreover, a temperature gradient is applied over the receptor layer which is monitored in time. The thermal resistance of the interface changes in function of the amount of target molecules bound to the receptor layer, blocking the heat flow through the interface and changing the gradient. It is a low-cost and homemade setup with a low detection limit (nM range) that has a wide range of applications. Additionally, it can be easily scaled down in terms of point-of-care sensing and any electrode that enables heat flow through IP layer can be incorporated into the device [10,178,223].

In food hazard detection, the only report to our knowledge utilizing HTM as readout platform employs a polyurethane bacteria-imprinted layer (Table 9). The samples were tested on apple juice samples and sensor exhibited a low limit of detection of 100 CFU/mL, which further emphasizes the application potential of the methodology.

## 6. Outlook and Conclusions

Currently, most industrial food plants still rely on external laboratories (some of them located abroad) for risk assessment of product’s safety. These laboratories make use of highly sensitive but expensive and time-consuming bench detection technologies (e.g., gas chromatography, PCR, media-based metabolic tests, etc.). During the testing time, companies need to either store the product until the results are obtained or distribute and recall the product in case of hazard detection, which translates to extra costs. Furthermore, developing countries and rural areas have no access to these technologies [224]. Consequently, there is a high demand of cost-effective and robust analytical devices to monitor food safety on-site, which would enable effective prevention and control strategies at the different processing steps.

In this context, an on-site testing device that is Affordable, Sensitive, Specific, User-friendly, Rapid and robust, Equipment free and Deliverable to end users (ASSURED) could have significant scientific and commercial impact [225]. IP-based technology is mainly interesting because of its affordable, user-friendly, robust nature and the fact it is equipment-free. The examples highlighted in the current review are often still in the laboratory phase of development, but, as technology becomes more advanced, they will get closer and closer to market penetration. In this respect, paper- and chip-based devices have gained a lot of interest as potential immobilization platforms for IPs in sensors that are able to detect food hazards in real samples. Paper-based platforms, for example, have been used for optical and thermal detection of pesticides, hormones and toxins [225,226,227,228] and are particularly interesting because of their low-cost, user-friendly mode of operation and equipment-free process. Another promising candidate for on-site testing are chip-based devices. They offer advantages such as small size, simplification of instruments, capability of multiplex assays, small amount of samples with precise control and high throughput analysis. They are a bit more expensive than paper-based assays, but they offer a higher degree of robustness. Chip-based devices have been combined with various read-out principles including colorimetric and SPR for the detection of the whole range of food contaminants, from drugs to insecticides and even whole-cell bacteria [199,228,229]. Devices such as these could accelerate the application of imprinting technology in the food-safety value chain from a technological perspective.

IPs also have a large benefit from a chemical and logistical perspective as they have an improved performance compared to their biological analogues in terms of shelf life, resistance to matrices (pH, ions, oxidizing conditions, etc.), LOD and response time. Additionally, traditional antibody-based sensors are more expensive because they require animals for the antibodies production. Animal storage, target compound isolation and purification, animal inoculation, purification and storage of the antibodies are all costs that IPs do not have. Therefore, the overall cost of producing IPs sensors is cheaper and it can be easily scaled up or down depending on the customers need. Despite these advantages and the fact that they are low-cost, user friendly-devices, no commercial IP-based sensors have been developed until date. One of the major issues yet to be overcome is the large-scale production of homogenous batches of IPs.

New advances in commercial MIP production could overcome this bottleneck in the future. Traditionally, commercial IP synthesis by big chemical companies such as Sigma-Aldrich mainly focuses on the development of IPs for separation purposes. In recent years, new players have entered the market such as MIP Diagnostics Ltd. They focus on designing homogenous batches of high-affinity MIPs specifically designed for biosensing. By collaborating with partners focusing on the integration of these MIPs in technically advanced readout platforms, huge steps towards valorization can be made in coming years [230,231]. Furthermore, in a globalized food chain, it has proven to be challenging to prevent foodborne outbreaks, and finally consumers are starting to get more interested in knowing how safe the food that they consume is. Easy IP-based test kits (e.g., colorimetric paper-based strips) could provide consumers more confidence in the food that they buy. IP technology can offer the consumer a way to accurately determine if their food is spoiled or contains compounds outside the regulatory limits, rather than having to trust the expiry date. In addition, such technology could also prevent food waste as a lot of food products are thrown away because the expiry date has passed while there might be nothing wrong with them. The work by Sergeyeva et al. illustrates what such a sensor might look like [186]; imprinted membranes can be designed for the detection of spoilage indicators and attached to food packaging. Rebinding of the indicator to the MIP membrane will then lead to a signal that the customer can read-out using, for instance, a smartphone. Similar studies further emphasize the potential impact of such a sensor on the food industry of the future [232,233]. In conclusion, further research on similar on-site IP-based test kits could lead to the development of smart sensing tools that are beneficial for every member belonging to the farm-to-fork production chain in coming decades.

## Figures and Tables

**Table 5:** Summary of recent publications for the recognition of bacteria using IPs in food samples.

Analyte (s)	Template/Monomer(s)/Crosslinker	IP Preparation	Food Sample	LoD	Readout Technique	Ref.
*Listeria monocytogenes*	*L. monocytogenes*/TRIM/DMA	Thermal: Pickering emulsion	Milk, pork	1 × 10^3^ CFU/mL	Optical	[197]
*Salmonella paratyphi*	*S. paratyphi*/MAH/EGDMA	Photo: Micro-contact	Apple juice	1.4 × 10^6^ CFU/mL	Optical	[199]
*Escherichia coli*	*E.* coli/MAH, HEMA/EGDMA	Photo: Micro-contact	Apple juice	1.5 × 10^6^ CFU/mL	Optical/ Acoustic wave	[200]
*Escherichia coli*	*E.coli*/4,4′-MDI, PG/BPA	Thermal: Micro-contact	Apple juice	1 × 10^2^ CFU/mL	Thermometric	[198]
*Escherichia coli*	*E.* coli/Dopamine	Electrochemical: Deposition	Water	8 CFU/mL	Electrochemical/optical	[77]
*Pseudomonas aeruginosa*	*P. aeruginosa*/Pyrrole	Electrochemical: Deposition	Apple juice	1 × 10^3^ CFU/mL	Electrochemical	[80]

TRIM, Trimethylopropane trimethacrylate; DMA, N, N-dimethylaniline; MAH, acrylate N-methacryloyl L-histidine methyl ester; EGDMA, ethylene glycol dimethacrylate; 4,4′-MDI, Diisocyanatodiphenylmethane; PG, Phloroglucinol; BPA, Bisphenol A.

**Table 6:** Imprinted polymer-electrochemical sensors for food safety.

Analyte	Sensor Composition IP/Electrode	Food Sample	Limit of Detection	Response Time	Ref.
Melamine	Poly (aniline-*co*-acrylic acid)/GCE	Milk	17.2 pM	20 min	[210]
Phosalone	Poly (APTES-*co*-TEOS)/Pt-UiO-66,CPME	Lake water, soil, wheat, cucumber orange	78 pM	2 min	[114]
Carbendazim	Poly(*O*-phenylenediamine)/S-Mo_2_C, GCE	Grape, apple, tomato, eggplant, cucumber	0.67 pM	6 min	[117]
Tetracycline	Poly (*P*-aminothiophenol)/Au	Honey	0.22 fM	30 min	[208]
Melamine	Polyaniline, Au/GCE	Milk, feed	1.39 pM	40 min	[211]
Diethylstilbestrol	Poly (APTES-*co*-TEOS *co*-OTOMS)/AuNP, MWCNT, GCE	Milk	24.3 fg/mL	15 min	[212]
Fumonisin B1	Poly (MAA-*co*-EGDMA)/GO-CdS, ITO	Milk, maize meal	4.7 pg/mL	15min	[213]
Fumonisin B1	Poly (MAA-*co*-EGDMA)/Ru, SiO_2_, CS, Au NP, GCE	Milk, maize meal	0.35 pg/mL	15 min	[214]
*Escherichia coli*	Poly (MAH-*co*-HEMA-*co*-EGDMA)/Au	Apple juice	70 CFU/mL	Real-time	[209]

GCE, Glassy carbon electrode; APTES, (3-aminopropyl)triethoxysilane, TEOS, Tetraethyl orthosilicate; Pt-UiO-66, metal–organic framework catalyst; CPME, Carbon paste microelectrode; Au, gold; SG, Sulfonated graphene; MWCNT, Multi-walled carbon nanotubes; OTOMS, Octyltriethoxysilane; MAA, Methacrylic acid; SPE, Screen printed electrode; CS, Chitosan; NP, Nanoparticles; EGDMA, Ethylene glycol dimethacrylate; GO, Graphene oxide; ITO, Indium tin oxide; MAH, N-methacryloyl-L-histidine methylester; HEMA, 2-Hydroxyethyl methacrylate.

**Table 8:** Imprinted polymer-piezoelectric sensors for food safety.

Transducer	Analyte	Sensor Composition IP/Electrode	Food Sample	Limit of Detection	Response Time	Ref.
*SPR/QCM*	*Escherichia coli*	Poly(HEMA-*co*-EGDMA-*co*-MAH)	Apple juice	3.72 × 10^5^ CFU/mL	56 s	[200]
*SPR*	Tobramycin	Poly (HEMA-*co*-MAGA)	Chicken egg white, milk	5.7 pM	Real time	[222]
QCM	Methimazole	Poly(MAA-*co*-EGDMA),silica	Pork, beef and milk	3 μg/L	8 min	[25]
QCM	Estradiol	Poly (MAA-co-Vinylpyrollidone-DHEBA)	Bread	2 μg/L	5–10 min	[23]
QCM	Trichlorfon (TCF)	Poly (Vinylidene difluoride)	Lettuce	15.77 ppb	6 h	[115]
QCM	Metolcarb	Poly(MAA-*co*-EGDMA)	Apple juice, pear, cabbage	2.309 μg/L	12 min	[112]

PEGDA, Poly (ethylene glycol) diacrylate; MAA, Methacrylic acid; EGDMA, Ethylene glycol dimethacrylate; QD, Quantum dots; MAH, N-methacryloyl-L-histidine methylester; HEMA, 2-Hydroxyethyl methacrylate.

**Table 9:** Imprinted polymer-thermometric sensors for food safety.

Analyte	Sensor Composition IP/Electrode	Food Sample	Limit of Detection	Response Time	Ref.
*Escherichia coli*	Polyurethane	Apple juice	100 CFU/mL	Real time	[198]

## Data Availability

Not applicable.
